# Myocardial Injury Complicated by Systolic Dysfunction in a COVID-19-Positive Dog

**DOI:** 10.3390/ani11123506

**Published:** 2021-12-08

**Authors:** Giovanni Romito, Teresa Bertaglia, Luigi Bertaglia, Nicola Decaro, Annamaria Uva, Gianluca Rugna, Ana Moreno, Giacomo Vincifori, Francesco Dondi, Alessia Diana, Mario Cipone

**Affiliations:** 1 Department of Veterinary Medical Sciences, Alma Mater Studiorum—University of Bologna, 40064 Bologna, Italy; giovanni.romito2@unibo.it (G.R.); f.dondi@unibo.it (F.D.); mario.cipone@unibo.it (M.C.); 2 Clinica Veterinaria Santa Teresa, 41032 Cavezzo, Italy; tere_@hotmail.it (T.B.); luigibertaglia@virgilio.it (L.B.); 3 Department of Veterinary Medicine, University of Bari, 70010 Valenzano, Italy; nicola.decaro@uniba.it (N.D.); annamaria.uva@uniba.it (A.U.); 4 Istituto Zooprofilattico Sperimentale della Lombardia e dell’Emilia Romagna “Bruno Ubertini”, 25124 Brescia, Italy; gianluca.rugna@izsler.it (G.R.); anamaria.morenomartin@izsler.it (A.M.); 5 Istituto Zooprofilattico Sperimentale dell’Abruzzo e del Molise “G. Caporale”, 64100 Teramo, Italy; g.vincifori@izs.it

**Keywords:** SARS-CoV-2, myocarditis, dilated cardiomyopathy phenotype, echocardiography, canine

## Abstract

**Simple Summary:**

Severe acute respiratory syndrome coronavirus 2 (SARS-CoV-2), the causative agent of the coronavirus disease 2019 (COVID-19) pandemic, is continuing to spread worldwide. As with many emerging infectious diseases, COVID-19 is of zoonotic origin, meaning that animals are susceptible to infection, including domestic pets such as dogs. Despite epidemiological surveys conducted in dogs living either in SARS-CoV-2-positive households or in geographic areas affected by COVID-19 steadily increasing, clinical reports aimed at characterising disease manifestation are currently scant in this species. This case report accurately describes the development of myocardial injury complicated by left ventricular systolic dysfunction in a SARS-CoV-2-positive dog. Interestingly, the clinical picture described herein closely resembles the cardiological compromise documented in SARS-CoV-2-positive humans and can therefore contribute to filling the current knowledge gap that exists between human and veterinary medicine concerning COVID-19.

**Abstract:**

A six-year-old Cavalier King Charles spaniel was referred with a two-month history of severe exercise intolerance and syncope. Clinical signs had developed during a local wave of coronavirus disease (COVID-19) two weeks after its family members had manifested symptoms of this viral disease and their positivity to severe acute respiratory syndrome coronavirus 2 (SARS-CoV-2) was confirmed. Cardiologic assessment documented myocardial injury complicated by systolic dysfunction. An extensive diagnostic work-up allowed us to rule out common causes of myocardial compromise, both infective and not. Accordingly, serological and molecular tests aimed at diagnosing SARS-CoV-2 infection were subsequently performed, especially in light of the dog’s peculiar history. Results of such tests, interpreted in the light of previous findings and current knowledge from human medicine, supported a presumptive diagnosis of COVID-19-associated myocardial injury, a clinical entity hitherto poorly described in this species.

## 1. Introduction

Since its emergence in December 2019 in Wuhan (China), severe acute respiratory syndrome coronavirus 2 (SARS-CoV-2), the causative agent of coronavirus disease 2019 (COVID-19), has spread worldwide in a short span of time [1]. Although such zoonotic disease has probably disseminated mainly by human-to-human transmission, the existence of hundreds of millions of companion animals living closely with humans raises the question of their potential role in the outbreak [1,2,3,4]. Accordingly, several studies have been recently performed in dogs and cats in many countries [2,3,4,5,6,7,8,9,10,11,12,13,14,15,16,17,18,19,20,21], demonstrating their susceptibility to this infection. However, it is important to acknowledge that the majority of these studies are serological and/or molecular surveys primarily aimed at evaluating the transmission of SARS-CoV-2 among domestic animals, not the related clinical compromise [5,6,7,8,9,10,11,12,13,14,15,16,17,18]. Consequently, to date, there is little evidence to determine how commonly SARS-CoV-2 naturally infected dogs and cats develop clinical signs and to characterise the clinical manifestations of the viral infection in these species. Given the above, clinical reports are needed, as they would help veterinarians in correctly interpreting findings from pets that have developed overt signs during a COVID-19 wave and/or have tested positive for SARS-CoV-2. In line with this need, this work aimed to report the development of myocardial injury (MI) complicated by left ventricular (LV) systolic dysfunction in a COVID-19-positive dog, a clinical picture poorly documented in this species [19,21], but well characterized in humans with SARS-CoV-2 infection [22,23,24].

## 2. Materials, Methods and Results

### Case Description and Clinical Investigations

A six-year-old, 9.7 kg, male Cavalier King Charles spaniel was referred to the Cardiology Unit of the Veterinary Teaching Hospital of the University of Bologna with a two-month history of severe exercise intolerance associated with a syncopal episode. Clinical signs had developed during a local wave of COVID-19 approximately two weeks after the family of its owner had manifested symptoms of this viral disease and their positivity to SARS-CoV-2 had been confirmed by the local Health authority. Despite the dog’s clinical condition, evaluation at our institution was postponed and performed only after two months from the occurrence of the aforementioned signs due to the COVID-19 illness and related quarantine of the owners. The dog had been previously evaluated by the primary veterinarian several times since he was a puppy, as regular examinations were performed approximately every six months. Previous medical history was unremarkable and no cardiac problems had been identified at earlier examinations. The patient was an indoor dog that was being fed a high-quality balanced commercial diet. He had no known exposure to toxic agents or medications and was current on vaccinations and parasite prevention.

**Figure 1:**
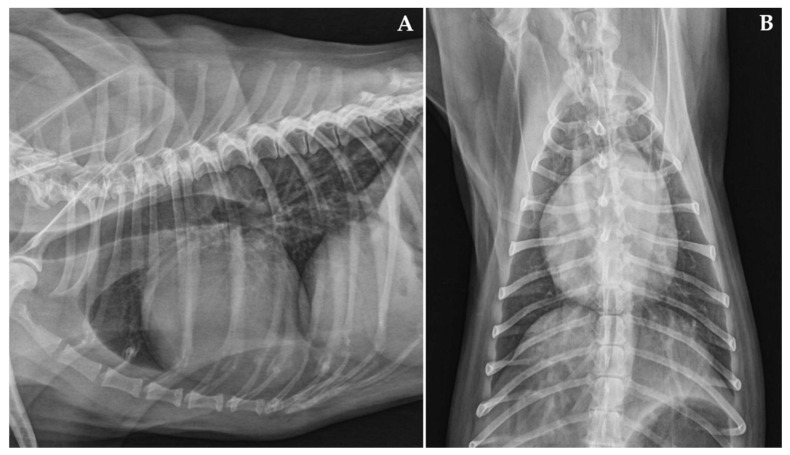
Right lateral (**A**) and dorso-ventral (**B**) radiographs of the thorax. Mild enlargement of the cardiac silhouette with no lung parenchymal abnormalities is evident.

**Figure 2:**
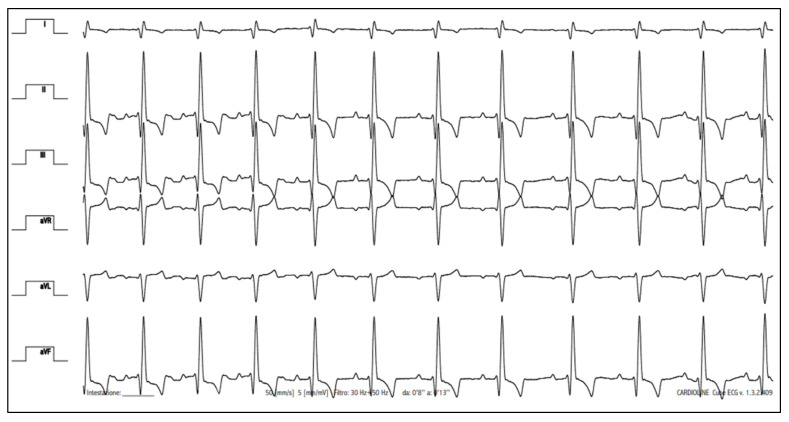
Six-lead electrocardiographic tracing. Sinus rhythm is evident. Paper speed = 50 mm/s; 1 cm = 2 mV.

**Figure 3:**
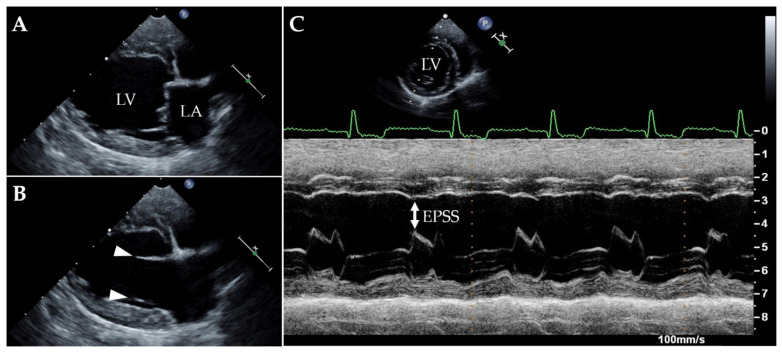
Two-dimensional echocardiographic findings obtained from a right parasternal long-axis four-chamber with open and closed mitral valve leaflets ((**A**) and (**B**), respectively). Note that the left ventricle is characterized by eccentric hypertrophy and a roundish appearance. Note also the lack of mitral valve abnormalities, as neither systolic prolapse nor valvular nodules can be identified (white arrowheads indicated leaflets during ventricular diastole). M-Mode echocardiographic findings obtained from a right parasternal short-axis view at the mitral valve level (**C**). Note the left ventricular systolic hypokinesia, expressed as a significantly reduced excursion of the left ventricular walls during systole and as a remarkable increase of the EPSS (white arrow). EPSS, mitral-valve E-point-to-septal-separation; LA, left atrium; LV, left ventricle; P: reference transducer mark; X over a white bar: focal point at the depth level of interest on the ultrasound image.

Upon presentation, cardiac auscultation revealed a grade II/VI left apical systolic murmur; the heart rate was 136 beats/min and the cardiac rhythm was regular. The femoral pulse was strong and synchronous with the heartbeat. Non-invasive systolic arterial blood pressure, assessed by a high-definition oscillometric device (petMAP graphic, Ramsey Medical, Inc., Tampa, USA), was 166 mmHg. Given the patient’s anxiety during physical examination, the pressure value was primarily interpreted as situational hypertension. Respiratory rate was mildly accelerated (44 breaths/min), likely due to the dog’s emotional stress, but lung auscultation was within normal limits. The remainder of the physical examination were unremarkable. Thoracic radiographs revealed mild generalised enlargement of the cardiac silhouette (vertebral heart scale 11.5, breed-specific reference interval 10.60 ± 0.50 [25]), with no obvious lung parenchymal abnormalities (Figure 1). Sinus rhythm was observed on a six-lead surface electrocardiogram (Cube ECG, Cardioline S.p.A., Caverano, Italy) (Figure 2). A transthoracic echocardiography was also performed by a board-certified cardiologist (GR) using an ultrasound unit (iE33 ultrasound system, Philips Healthcare, Monza, Italy) equipped with phased-array transducers (3–8 and 1–5 MHz) and continuous electrocardiographic tracing (Figure 3, Supplementary Materials Videos S1–S4). This showed LV volume overload and global systolic dysfunction without concomitant left atrial dilation (Table 1). Although the mitral valve leaflets were structurally and functionally normal, a mild mitral regurgitation with central jet was present. In light of the aforesaid findings, the valve insufficiency was hypothesized to be functional and due to the dilated cardiomyopathy (DCM) phenotype. No other echocardiographic abnormalities were identified.

Results of routine blood work, including complete blood count, serum chemistry and coagulation profile (prothrombin time, activated partial thromboplastin time, fibrinogen and antithrombin), were unremarkable. In light of the DCM phenotype, further laboratory tests included a thyroid profile and assessment of the serum concentration of cardiac troponin I (cTnI, IMMULITE 20000, Siemens, Erlangen, Germany). The former test ruled out hypothyroidism as a possible cause of LV systolic dysfunction (thyroxine 29.5 nmol/L, hospital reference interval [HRI] 13–51 nmol/L; thyroid stimulating hormone 0.12 ng/mL, HRI 0.03–0.38 ng/mL), while the latter unveiled MI (0.19 ng/mL, HRI < 0.15 ng/mL) [26,27]. The dog was started on pimobendan (Vetmedin, Boehringer Ingelheim, Ingelheim amRhein, Germany) at a dose of 0.25 mg/kg orally every 12 h, and several blood samples were collected with the aim of submitting them for investigation of infections responsible for MI. Initially, serological tests for *Borrelia burgdorferi*, *Dirofilaria immitis*, *Anaplasma phagocytophilum*, *Ehrlichia canis*, *Leishmania infantum*, *Toxoplasma gondii* and *Bartonella henselae* infection were performed (SNAP 4Dx, IDEXX Laboratories, Inc., Westbrook, U.S.A.; MegaFLUO LEISH, Vetefarma S.r.l., Cuneo, Italy; MegaFLUO TOXOPLASMA Gondii, Vetefarma S.r.l., Cuneo, Italy; Indirect immunofluorescence performed as described by Fabbi et al. [28]), yielding negative results. Given the family history as well as the ongoing and still unexplained MI, further serum samples were subsequently submitted for SARS-CoV-2 investigation [19,21]. Different serological assays were performed (using samples collected on the same day) to detect antibodies against the S and the N proteins of SARS-CoV-2. More in detail, specific neutralizing antibodies against the receptor binding domain of the spike protein were determined in serum samples using the SARS-CoV-2 surrogate virus neutralisation test (sVNT, GenScript cPass™ SARS-CoV-2 Neutralisation Antibody Detection Kit, GenScript Biotech Co., Ltd., Leiden, Netherlands) following the manufacturer’s instructions (positivity: ≥30% inhibition). Furthermore, SARS-CoV-2 neutralization assay (VNT) was performed as described by Rijkers et al. [29] (positivity: titers ≥1/10). Lastly, a commercial multispecies enzyme-linked immunosorbent assay (ELISA; EradikitTM COVID19-Multispecies, In3Diagnostic, Turin, Italy; positivity: >20%) was performed. Pending serological results, no other therapies were prescribed in addition to pimobendan, but exercise restriction was recommended. Within two weeks, results from serological assays became available. All tests yielded a positive result (Table 2). 

In light of such findings, approximately two weeks from presentation, another control was performed to recheck the clinical, cardiological and serological condition of the dog, and to obtain nasopharyngeal and rectal swabs for SARS-CoV-2 genome detection (using a real-time polymerase chain reaction (PCR) targeting E gene, as previously described by Corman et al. [30]). The owner reported an improvement in exercise tolerance and no further syncopal episodes. Physical evaluation was unremarkable, with the only exception being the heart murmur, which maintained unchanged characteristics. Echocardiography demonstrated a mild improvement of LV systolic function, although a DCM phenotype and a functional mitral regurgitation were still present, thus explaining the persistence of the heart murmur (Table 1). Blood samples were collected to monitor the antibodies against SARS-CoV-2 as well as the serum concentration of cTnI. All serologic assays were still positive (Table 2) and cTnI was still over the HRI (0.17 ng/mL). SARS-CoV-2 molecular analysis yielded a negative result. Therapy as well as instruction at home were unchanged, and another recheck was planned within three weeks. 

At that control, although the clinical and echocardiographic findings were stable compared to the previous examination, and two serological tests were still positive (i.e., sVNT and VNT), cTnI was normalised (0.09 ng/mL) and ELISA yielded a negative result for SARS-CoV-2 (Table 2). The dog continued to receive pimobendan and be revaluated regularly over the following weeks, showing a good clinical condition, stable echocardiographic parameters and a cTnI within the HRI. He is still alive and doing well at the time of manuscript writing (9 months from the occurrence of clinical signs and 7 months from the first evaluation at our institution).

## 3. Discussion

This report describes a COVID-19-positive dog with MI and LV systolic dysfunction. Left ventricular systolic dysfunction was suspected as a possible consequence of MI since the breed was not typical for primary DCM and the dog’s history made other secondary causes of a DCM phenotype—namely nutritional and drug-induced myocardial dysfunction, hypothyroidism, tachycardia-induced cardiomyopathy and post-resuscitation myocardial dysfunction [31,32,33]—unlikely.

In dogs, the term MI is conventionally used for subjects in which at least one cardiac troponin concentration, especially cTnI, is above the upper reference limit [26,27]. Both infectious and non-infectious diseases can cause MI in this species [25,26,32,33,34]. Among infectious triggers, bacteria (e.g., *Bartonella* spp., *B. burgdorferi* and *E. canis*) and parasites (e.g., *L. infantum* and *D. immitis*, *T. gondii*) represent oft-cited causes of canine MI, especially in adult dogs, whereas viruses seem to trigger MI more commonly in puppies (e.g., canine parvovirus and canine distemper virus) [26,34,35]. Accordingly, the dog in this report was initially tested for several bacterial and parasitic diseases, purposefully researching those known to be present in Italy and capable of causing MI in this species [26,34,35,36]. As the dog tested negative for these pathogens and non-infectious causes of MI were considered unlikely based on the dog’s history, we subsequently started considering less common infective triggers of MI. In light of the peculiar timing of onset of clinical signs (i.e., during a local wave of COVID-19, soon after which the owners became symptomatic due to SARS-CoV-2), we considered testing the dog for this emerging viral disease. Such a choice was also supported by the fact that, at that time, a study had been published documenting myocardial compromise in pets naturally infected with COVID-19 [19]. Interestingly, our dog tested positive to different serological assays but negative to PCR performed on nasopharyngeal and rectal swabs. Knowledge of epidemiological and pathophysiological features of this viral disease in dogs is essential to properly interpret this result in light of the patient’s clinical picture. 

Concerning epidemiological data from our country, three large-scale Italian surveys conducted on domestic pets living either in SARS-CoV-2-positive households or in geographic areas severely affected by COVID-19 reported a seroprevalence in dogs of 1.1–3.3% [5,8,17]. Interestingly, in two of these studies, seroprevalence was higher among animals living in close contact with SARS-CoV-2-positive owners [5,8]. Moreover, among some serologically positive dogs from the survey by Colitti et al. [8], owners reported that their pets experienced clinical signs in proximity to the period during which they manifested COVID-19 illness. These findings appear to be in line with the history of our dog. Another consideration worthy of mention regarding the serological evaluation of pets affected by COVID-19 concerns the possible discrepancy of results between the various available tests. This has been recently demonstrated by Decaro et al. [18], who monitored the SARS-CoV-2 antibody response in 7 dogs and 2 cats by using two multispecies ELISA tests, plaque reduction neutralisation test and VNT [18]. Such a discrepancy may be related to a lower sensitivity of ELISA or, alternatively, to a lack of specificity of neutralization assays [18]. Thus, we decided to test our dog’s samples obtained at different collection time points by using several assays, in order to reduce the possibility of misinterpretation of laboratory results. In line with Decaro et al. [18], we found a partial discrepancy between different tests (i.e., at last sample analysis, ELISA was negative and the remaining serological tests were positive). Our results, interpreted in the light of the previous report [18], strengthen the recommendation to test samples from dogs suspected of being COVID-19-positive with multiple serological tests, as the decision to use a single assay may be associated with the risk of misdiagnosis.

In two of the aforesaid large-scale Italian surveys, both serological and molecular tests were performed [5,17]. Intriguingly, all animals tested by PCR gave negative results, including those animals living in households with confirmed COVID-19 human infection, those with positive serological results and those with clinical signs [5,17]. The absence of clearly positive PCR results in these studies is in line with findings from previous molecular investigations conducted in dogs from other European countries, North America and Asia [10]. Similarly, the discrepancy between serological and molecular results in this species has been documented in other studies [11,18,19]. This finding is likely related to the limited time span of virus shedding combined with the relatively long duration of circulating antibodies after exposure. For example, in experimentally infected dogs, the virus has been detected in faeces up to six days post-infection, but not in oropharyngeal swabs [37], whereas naturally infected dogs may show seropositivity even beyond 2–10 months after exposure [8,18]. Therefore, delayed sampling of our dog (more than two months after the onset of the dog’s clinical signs because of restrictions on owner movement due to their quarantine) represented a likely explanation for the concomitant positive serology and negative PCR reported herein [38]. Similar temporal issues have been reported to complicate the interpretation of diagnostic tests even in humans affected with COVID-19 [39], as viral load can be undetectable 20 days after onset of clinical signs [40] but SARS-CoV-2 neutralizing antibodies can persist from 6–8 months to more than 12 months in this species [41,42,43,44]. In the present case, the PCR negativity, interpreted in the light of the dog’s history and clinical, cardiological and serological findings, did not preclude us from suspecting COVID-19 as a likely trigger of underlying myocardial compromise. Our hypothesis was strengthened not only be our extensive diagnostic work-up, which allowed us to exclude other differentials for MI and systolic dysfunction, but also by current knowledge from human medicine [39]. Specifically, as the SARS-CoV-2 molecular assay is subjected to viral load dynamics over time and false-negative results have been documented in affected humans, physicians recommend to interpret COVID-19 test laboratory results in the overall context of each patient’s clinical presentation, as well as not to exclude SARS-CoV-2 only in the light of a negative result by a single laboratory assay [39,45,46]. Such a recommendation becomes particularly important, especially in patients showing highly suggestive history and clinical signs for SARS-CoV-2 but negative results to PCR [39].

Concerning COVID-19 pathogenesis and the related clinical compromise, respiratory symptoms represent the most common manifestation of the disease in humans [1]. Similarly, respiratory signs are overrepresented among the few symptomatic dogs hitherto reported [6,8,11]. However, as COVID-19 is a polymorphic disease characterised by great variability in clinical presentation, non-respiratory complications may sometimes occur [19,20,21]. In humans, cardiac involvement during SARS-CoV-2 infection has been documented by many authors and represents a source of great concern [22,23,24]. The mechanisms that lead to cardiac involvement in the setting of COVID-19 include direct injury caused by direct viral entry to cardiomyocytes, which is possible by direct viral binding to the angiotensin-converting enzyme 2 present on these cells, hypoxia-induced myocardial ischaemia, and exaggerated inflammatory response characterised by endothelial overactivation and microvascular thrombi [22,23,24]. Histologically, this may trigger non-myocarditis inflammatory infiltrates, acute myocardial infarction, and, rarely, myocarditis [47,48], which represent the anatomical prerequisites for the development of clinical complications, such as LV systolic dysfunction, congestive heart failure and arrhythmias [22,23,24]. 

Similar cardiological complications have been documented in pets naturally infected with COVID-19, by Ferasin et al. [19,21]. Although these studies merit interest, as they represent the first two reports on possible cardiac involvement in COVID-19-positive dogs and cats, some drawbacks weaken their content. For example, contrary to our report, the study populations of the aforesaid investigations did not systematically undergo an extended diagnostic work-up aimed at excluding concomitant diseases capable of causing cardiovascular complications [19,21]. Therefore, it could be speculated that the SARS-CoV-2 infection was incidental and did not represent the primary cause of cardiac compromise in some animals. Another source of concern regards the terminology employed in the first reports of Ferasin et al., since the authors stated that the cardiac abnormalities of their study population were secondary to myocarditis [19]. As the clinical suspicion of myocarditis (namely, an inflammatory disease of the myocardium diagnosed by established histological, immunological and immunohistochemical criteria) necessarily requires histological confirmation [49], their statement can be considered inappropriate, since neither autoptic evaluation nor endomyocardial biopsy were performed by the authors [19]. As such diagnostic tests were not performed even in our case, we have purposefully discussed MI rather than myocarditis to be more consistent with proper scientific nomenclature [49,50,51]. Lastly, no echocardiographic measurement or images aimed at characterising the type and degree of myocardial compromise are available from the first of the two aforementioned studies [19], while echocardiographic images of a single positive cat were provided in the second one [21]. In contrast, we provided, for the first time in veterinary medicine, detailed echocardiographic data of a COVID-19-positive dog with MI.

This report has some limitations, including the above-mentioned lack of histopathology aimed at gaining further information on the type and extent of myocardial compromise. The lack of molecular analysis at the time of the onset of the patient’s clinical signs, inevitably related to restrictions on owner movement, represents another limit as it would have increased the chance to detect the viral genome. Theoretically, such a delay could have also contributed to a blunting of the cTnI values over the weeks following disease manifestation, as a result of a progressive myocardial recovery. This could explain the relatively mild increase of cTnI values observed on presentation to our institution. Lastly, as our dog was not tested for all the reported infective causes of myocarditis, it cannot be completely excluded that concomitant pathogens may have also contributed to MI. However, our patient tested negative for the more common infective causes of canine myocarditis reported in Italy; moreover, simultaneous detection of SARS-CoV-2 along with other cardiotropic pathogens during myocarditis represents an exceptionally rare condition in humans [51,52].

In conclusion, this report supports a role for SARS-CoV-2 as a causative agent of canine MI. Clinicians should be aware of the existence, echocardiographic features and clinical significance of cardiac involvement in COVID-19-positive dogs and consider this emerging disease in the list of triggers of MI and systolic dysfunction in this specie. Moreover, the case described herein represents an excellent example of the importance of interpreting tests aimed at detecting COVID-19-positivity using a holistic approach, considering all the available findings, including those from the patient’s history, physical examination, various laboratory assays and echocardiography. Lastly, this report highlights the importance of a multidisciplinary approach to the diagnosis and clinical management of such emerging viral disease in dogs, as previously reported in humans.

## Figures and Tables

**Table 1:** Selected echocardiographic findings measured at arrival (T0) and at first control (T1) in the dog from this report.

Parameter	T0	T1	Reference Intervals
LA/Ao	1.2	1.15	<1.6 ^a^
LAD (mm)	30	28	22.1–33.1 ^b^
LVIDDn	1.95	1.9	1.27–1.85 ^c^
LVIDSn	1.64	1.48	0.71–1.26 ^c^
EDVI (mL/m^2^)	148	126	49.8–122.4 ^d^
ESVI (mL/m^2^)	96	70	13.2–38.0 ^d^
SF (%)	16	22	30–49 ^d^
EF (%)	35	45	57.8–82.1 ^d^
EPSS (mm)	12	9	<6.5 ^e^

LA/Ao: left atrial-to-aortic root ratio; EDVi: end-diastolic volume index; EF: ejection fraction; EPSS: mitral-valve E-point-to-septal-separation; ESVi: end-systolic volume index; LAD: left atrial anteroposterior diameter; LVIDDn: left ventricular internal diameter in diastole indexed to body weight; LVIDSn: left ventricular internal diameter in systole indexed to body weight; SF: shortening fraction. ^a^ Rishniw, M.; Erb, H.N. Evaluation of four 2-dimensional echocardiographic methods of assessing left atrial size in dogs. *J. Vet. Intern. Med.* **2000**, *14*, 429–435. ^b^ Marchesotti, F.; Vezzosi, T.; Tognetti, R.; Marchetti, F.; Patata, V.; Contiero, B.; Zini, E.; Domenech, O. Left atrial anteroposterior diameter in dogs: reference interval, allometric scaling, and agreement with the left atrial-to-aortic root ratio. J. Vet. Med. Sci. **2019**, *81*, 1655–1662 (values expressed as minimum-maximum). ^c^ Cornell, C.C.; Kittleson, M.D.; Della Torre, P.; Häggström, J.; Lombard, C.W.; Pedersen, H.D.; Vollmar, A.; Wey, A. Allometric scaling of M-mode cardiac measurements in normal adult dogs. *J. Vet. Intern. Med.* **2004**, *18*, 311–321 (values expressed as 2.5th–97.5th 239 percentiles). ^d^ Serres, F.; Chetboul, V.; Tissier, R.; Poujol, L.; Gouni, V.; Carlos Sampedrano, C.; Pouchelon, J.L. Comparison of 3 ultrasound methods for quantifying left ventricular systolic function: correlation with disease severity and prognostic value in dogs with mitral valve disease. *J. Vet. Intern. Med.* **2008**, *22*, 566–577 (values expressed as minimum-maximum). ^e^ Holler, P.J.; Wess, G. Sphericity index and E-point-to-septal-separation (EPSS) to diagnose dilated cardiomyopathy in Doberman Pinschers. *J. Vet. Intern. Med.* **2014**, *28*, 123–129).

**Table 2:** Serological findings recorded at arrival (T0) and at first (T1) and second control (T2) in the dog from this report.

Serological Test	T0	T1	T2
sVNT	60%	66%	60%
VNT	1/20	1/10	1/10
ELISA	27.37%	22.55%	negative

ELISA: enzyme-linked immunosorbent assay; sVNT: surrogate virus neutralization test; VNT: virus neutralization test.

## Supplementary Materials

The following are available online at https://www.mdpi.com/article/10.3390/ani11123506/s1, Video S1: Two-dimensional transthoracic echocardiographic video clip obtained from a right parasternal long-axis four-chamber view. Note the left ventricular volume overload and systolic dysfunction. Video S2: Color Doppler transthoracic echocardiographic video clip obtained from a right parasternal long-axis four-chamber view. Note the mid-to-moderated mitral regurgitation with central jet. Video S3: Two-dimensional transthoracic echocardiographic video clip obtained from a right parasternal short-axis view at the papillary muscle level. Despite some artifacts due to the uncooperativeness of the patient and his emotional tachypnea, global left ventricular systolic dysfunction can be appreciated. Video S4: Two-dimensional transthoracic echocardiographic video clip obtained from a right parasternal short-axis view at the aortic root level. This view, combined with findings from Video S1, allow appreciating the lack of left atrial dilation.
